# Factors influencing adoption of sexual and reproductive health intervention for adolescents in Ebonyi, Nigeria

**DOI:** 10.1186/s12913-024-11103-y

**Published:** 2024-05-19

**Authors:** Aloysius Odii, Ifeyinwa Chizoba Akamike, Chinyere Ojiugo Mbachu, Obinna Onwujekwe

**Affiliations:** 1 Health Policy Research Group, University of Nigeria Nsukka, Enugu, Nigeria; 2 Department of Community Medicine, College of Medicine, University of Nigeria, Enugu, Nigeria; 3 Department of Community Medicine, Alex Ekwueme Federal University Teaching Hospital, Abakaliki, Ebonyi, Nigeria; 4 Department of Sociology/Anthropology, University of Nigeria, Nsukka, Enugu, Nigeria

**Keywords:** Enablers, Constraints, Adoption, Sexual and reproductive health services, Community-embedded

## Abstract

**Background:**

School and Community-embedded reproductive health interventions have been implemented in developing countries, with evidence that they led to improved sexual and reproductive health among adolescents. However, this type of intervention is rarely evaluated for its potential adoption and use. This study evaluated the constraints and enablers of the adoption of a school and community-embedded intervention that used community engagement, capacity building, partnerships and collaborations to deliver sexual and reproductive health services to adolescents.

**Methods:**

The intervention was implemented between 2019 and 2021 in six local government areas in Ebonyi State. The results on adoption presented here were collected four months into the mid-phase of the project, targeting adolescents, parents, adult family members, healthcare providers, local authorities, and community members. Sixteen in-depth interviews were conducted with policymakers, 14 with health service providers and 18 Focus Group Discussions (FGDs) with parents, community leaders and adolescents who were part of the implementation process. The coding reliability approach, a type of thematic data analysis was used, that involves early theme development and the identification of evidence for the themes.

**Results:**

The adoption of school and community-embedded reproductive health intervention was strong among stakeholders at the early stages of the implementation process. Multi-stakeholder involvement and its multi-component approach made the intervention appealing, thereby enabling its adoption. However, at the later stage, the adoption was constrained by beliefs and norms about sexual and reproductive health (SRH) and the non-incentivisation of stakeholders who acted as advocates at the community level. The sustainability of the intervention may be threatened by the non-incentivisation of stakeholders and the irregular supply of materials and tools to facilitate SRH advocacy at the community level.

**Conclusions:**

The inclusive community-embedded reproductive health intervention was adopted by stakeholders because of the enablers which include timely stakeholder engagement. However, for it to be sustainable, implementers must address the non-incentivising of community-level advocates which serve as constraints.

**Supplementary Information:**

The online version contains supplementary material available at 10.1186/s12913-024-11103-y.

## Background

Access to Adolescent Sexual and Reproductive Health (ASRH) services in sub-Saharan African countries is still sub-optimal. This is a public health challenge because of the increased risks of unwanted and unplanned pregnancies, and sexually transmitted infections among others [1, 2]. Adolescent sexual and reproductive health interventions have recently gained significant ground as a way to curb this challenge [3]. They aim to improve the usage of ASRH services thereby enhancing the coping mechanisms of adolescents, prevent unwanted pregnancy and sexually transmitted infections [4]. Furthermore, they seek to reduce the negative effects of sexual behaviour and enhance the quality of sexual relationships for young people, both now and in the future.

One such intervention used in enhancing ASRH is the Community-Embedded Reproductive Health Care for Adolescents (CERCA) that was developed, implemented and evaluated by Decat and his associates in Latin America cities (Cochabamba in Bolivia, Cuenca in Ecuador, and Managua in Nicaragua) [3, 5, 6]. Consequently, the project combined the creation of an enabling environment, the utilization of media, community engagement, and the involvement of policymakers to enhance adolescent competence in making reproductive health choices, with a particular focus on reducing unplanned pregnancies [7]. The CERCA project was successful because it sparked conversations about ASRH and responsibilities and encouraged continued community action [8]. The impact of the CERCA project underscores the need for its implementation in similar settings like Nigeria—a developing country grappling with multifaceted challenges in adolescent sexual and reproductive health.

The effectiveness of ASRH interventions has been proven and their benefits in improving the health and well-being of adolescents cannot be over-emphasised [9]. However, there still exists some level of apathy towards the implementation and adoption of adolescent sexual and reproductive health interventions despite its presence in the health agenda of several countries [4, 10].

Adoption, which is only one type of implementation research outcome, refers to the process of deciding to commit to using an intervention or policy [11]. Other implementation research outcomes include acceptability, appropriateness, feasibility, fidelity, implementation cost, penetration, and sustainability [12]. Adoption can be said to be an intention, initial decision, or action to apply or utilize an innovative and proven practice or intervention [12, 13]. We focused on adoption given its significance in the integration and scale-up of interventions. Public health interventions rely not only on their efficacy but also on successful adoption [14].

Previous studies have highlighted a number of enablers and constraints to the adoption of health interventions, including ASRH interventions [11]. Some identified enablers of adoption of intervention include stakeholder involvement, and supervision, while constraints include financial constraints, delay in supply of equipment, and shortage of staff [9, 15]. Political support, perceived need and benefits of innovation, compatibility, adaptability, availability/quality of resources, availability of financial resources, integration of new programming, shared vision, shared decision making, coordination with other agencies, communication, and supervisory support have also been reported as factors affecting the adoption of interventions [11]. These studies all have in common the evaluation of barriers and facilitators, but not specific to the adoption of a community-embedded SRH intervention.

Engaging communities is effective in reducing risks to sexual and reproductive health in Nigeria [16]. However, community support and building the capacity of stakeholders are paramount if interventions will work [17]. Likewise, there is a call for an innovative approach that institutionalizes sex education in schools and communities [18]. The adoption of these approaches could help governments and communities become more aware of SRH issues and motivated to work together to improve adolescent health. Therefore, understanding the factors affecting the adoption of community-embedded adolescent sexual and reproductive health intervention is critical in ensuring that intervention leads to integration and possible scale-up to other communities and countries.

While there is evidence that an inclusive community-embedded reproductive health intervention improves access to quality SRH services for adolescents, evidence of the adoption of this approach is scarce [3]. This study intends to fill the gap by exploring the enablers and constraints to the adoption of an inclusive community-embedded intervention for delivering SRH services to adolescents in Nigeria. Adoption was measured from the perspective of policymakers, providers, community members, and adolescents who participated in the implementation process [12].

## Conceptual framework

**Fig. 1:**
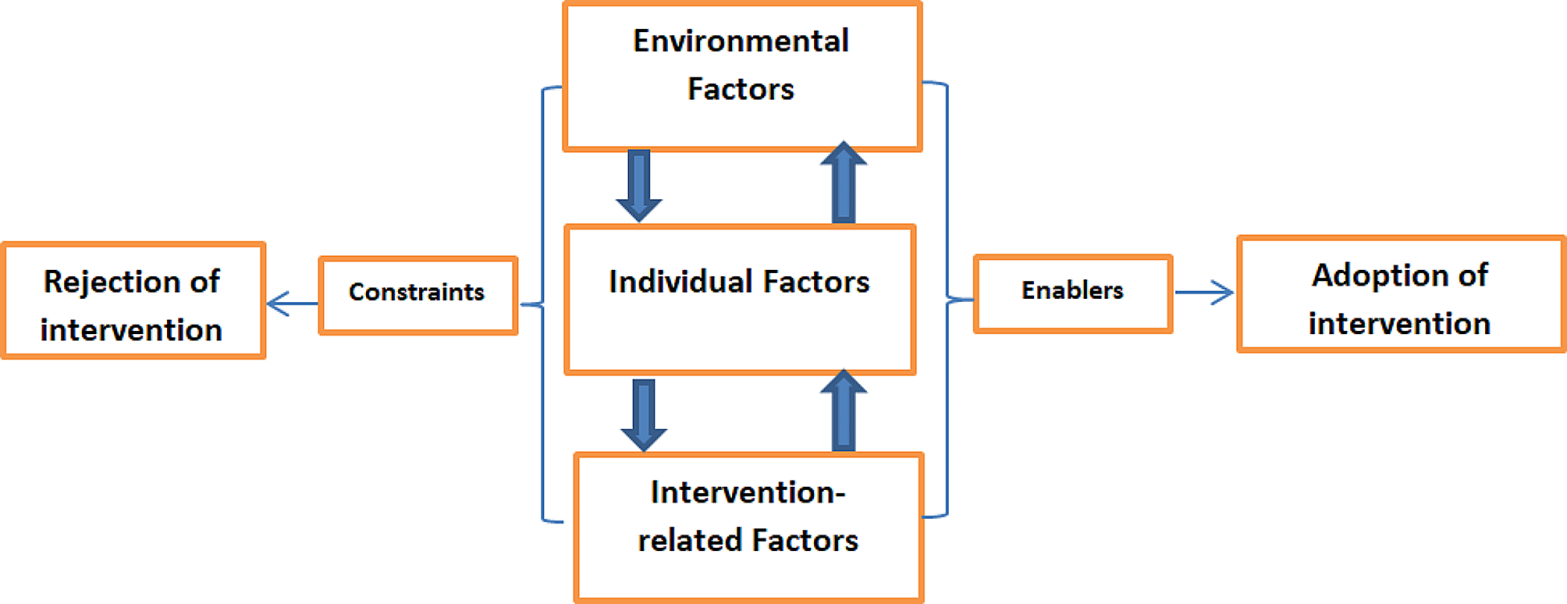
Conceptual framework showing the factors (enablers and constraints) affecting the adoption of community-embedded ASRH intervention

For the purpose of this study, we mapped out three key factors to elucidate both constraining factors and enablers influencing adoption: environmental factors, individual factors, and intervention-related factors (Fig. 1). These factors were conceptualized based on the ecological framework, which highlights community-level factors, provider characteristics, and intervention attributes. Environmental factors encompass the broader contextual elements surrounding program implementation, such as community dynamics and contextual complexities. Within this domain, certain factors may act as enablers, facilitating adoption, while others may serve as constraints, impeding progress. Similarly, individual factors relate to the personal attributes, beliefs, and behaviors of participants involved in the adoption process. These factors could either foster adoption or present as barriers to implementation. Intervention-related factors encompass the intrinsic characteristics of the program or innovation, outlining its unique features and functionalities. Also, certain intervention factors may enhance adoption, while others may pose challenges. Environmental factors may influence individual factors, which, in turn, may interact with intervention-related factors. Conversely, intervention-related factors could impact both environmental and individual factors. It’s important to note that when these factors serve as constraints, they hinder adoption, whereas when they act as enablers, they promote adoption of intervention [19].

## Methods

### Study design and area

This study was a qualitative study embedded in a larger intervention study that implemented school and community-based ASRH interventions in communities in Ebonyi State, between 2019 and 2021 [20]. Ebonyi State is located in the South Eastern region of Nigeria. The state is made up of 13 local government areas (LGAs) divided into three senatorial zones. The state is known for its agricultural activities and is also known to be a source of some solid minerals. Compared to other south-eastern states in Nigeria, Ebonyi has poor ASRH indices – (an unmet need for family planning of 23% and contraceptive prevalence rate of 8.2%) [21].

Twelve communities were selected from six LGAs (two per LGA) with six [6] receiving adolescent sexual and reproductive health (ASRH) interventions while the remaining six [6] served as non-intervention communities. LGAs with high unmet needs, evidenced by unwanted teenage pregnancies and abortions, were prioritized, ensuring a selection that reflects both rural and urban spread [22]. The intervention lasted for 2 years, it comprised (i) advocacy to stakeholders (ii) training of teachers, peer educators, and health service providers, and (iii) small group awareness campaign sessions.

The researchers adapted the CERCA community-embedded intervention to address the SRH needs of adolescents. The approaches are described below:

### Advocacy to stakeholders

Advocacy visits to influential decision-makers in the state were carried out by the researchers, led by OO. This lasted for two consecutive weeks, especially on weekdays. The visit aimed to gain their support for the project and to encourage pro-adolescent SRH decision-making and policy changes at local and state levels. Influential decision-makers were selected based on their position in ministries or agencies that have some impact either directly or indirectly on sexual and reproductive health of adolescents. A letter of introduction was drafted and forwarded to the target agencies, leading to the scheduling of appointments. The targeted agencies for advocacy visits were Ebonyi State Ministry of Health, Ebonyi State Primary Health Care Development Agency, Office of the Sustainable Development Goals, Ebonyi State Ministry of Education, Universal Basic Education Board, Ebonyi State Ministry of Information & Culture, Ebonyi State Broadcasting Corporation, Ebonyi State Ministry of Youth and Sports Development, Agency for Mass Literacy and Non-formal Education. Two legislators – house committee chairmen on health and education were also visited. During each advocacy visit, the advocacy team made a presentation of key findings from the situation analysis on adolescent sexual and reproductive health in Ebonyi state and an appeal for political support in institutionalizing comprehensive sex education for adolescents in secondary schools and non-formal education in selected communities in Ebonyi state.

The community leaders were also visited. The advocacy team leveraged the monthly meeting of the Council of Traditional Rulers (CTR) to present key findings from the situation analysis on sociocultural influences on adolescent SRH and the roles of traditional rulers in addressing sociocultural barriers to accessing SRH information and services for adolescents [23–25]. Support was also sought from traditional rulers in implementing the intervention. This primarily involved seeking their assistance in community engagement, cultural guidance, and advocacy. Thereafter, the team visited the six study communities and interacted with community leaders (including leaders of women and youth groups, religious leaders and town union executives).

The contacts with stakeholders shaped the development/implementation of the intervention. Some of the stakeholders were included as boundary partners (LGA adolescent focal persons, trained school principals, trained teachers, and peer health educators) in the implementation and subsequently interviewed.

### Training of health providers, teachers, and peer educators

A three-day residential training workshop was carried out for senior and mid-level healthcare managers who provide comprehensive and adolescent-friendly SRH services. The participants were drawn from the six LGAs in Ebonyi State. The aim was to build their capacity as providers. Before the training, participants were offered a pre-test to assess their knowledge, followed by another test at the end of the exercise. A total of 29 participants were trained. The training manual used consisted of eight modules: (i) introduction to adolescence and adolescent health; (ii) sexuality and sexual behaviours; (iii) sexually transmitted infections; (iv) principles and practice of counselling; (v) Pregnancy and prevention of pregnancy; (vi) counselling practices on selected health issues of adolescents (including values clarification); (vii) optimal adolescent and youth-friendly services; and (viii) record-keeping and health information systems. The trained providers then stepped down the training to frontline service providers including PHC workers, community health workers and patent medicine vendors through a series of training workshops. In total, 80 voluntary CHWs, and 80 PMVs were trained. More details about the training formats can be found here [26].

Teachers and peer educators were also trained in the provision of sexual and reproductive health information to adolescents and this lasted for two days. Two workshops (one for teachers and another for peer health educators) were held concurrently. The training manual had eight modules namely: (i) puberty and pubertal changes; (ii) sexual abstinence; (iii) premarital and teenage pregnancy; (iv) human sexual behaviour and sexually transmitted infections; (v) contraception and safe sex; (vi) sex and gender roles, including issues of gender-based violence and sexual abuse; (vii) sources of sexual and reproductive health information and services; and (viii) effective parent-child communication of SRH. Teachers alone also received training on an additional module on principles and practice of counselling. Both teachers and students had a combined session on the last day to learn and discuss the process of establishing school health clubs.

### Awareness campaign sessions

A total of five sessions were organised in each implementing LGA with a maximum of 20 persons in each session. A session with parents of adolescents in the community, one session with community leaders (such as religious leaders, traditional leaders, village heads, youth leaders, women leaders, men leaders and leaders of security personnel in the community) and three different sessions with adolescent boys and girls who are either in school or out of school. For parents and community leaders’/influencers’ sessions, information focused on ASRH (Basic information on effective parent-child communication about ASRH matters, basic principles and benefits of good parent-child communication skills, things to avoid and effects of poor parent-child communication on ASRH matters). Other components of the intervention included the distribution of the campaign materials (posters, handbill, face-caps, T-shirts, hand bands etc.) and, pasting of posters (designed specifically for adolescents and parents) in the communities.

Separate sessions were organized for adolescent boys and girls. Trained school teachers, peer educators, and adolescent health focal officers facilitated sessions with adolescents and adults, with technical support provided by the researchers. They were provided with stipends to cover transport fares. Additionally, some young adults who have interest and passion were selected to serve as mentors to adolescents in the community. They were identified through self-expression and active engagement in the activities. Their primary role involves helping them build confidence in making decisions and referring or accompanying them to appropriate healthcare providers. Adolescents engaged in discussions covering various topics, including adolescent sexuality, sexual and reproductive health (SRH) rights, abstinence, contraceptive methods to prevent unintended pregnancies, life skills to prevent sexual violence and exploitation, reporting procedures for sexual assault, accessing SRH information and services, and strategies for preventing and reporting COVID-19 infections.

### Study participants and sampling

Four months into the mid-phase of implementing the intervention, a qualitative evaluation was done to assess the adoption. The study participants comprised in-school and out-of-school adolescent boys and girls who are between 13-to-18-years, parents/caregivers of these adolescents, community leaders, teachers, and policymakers. Participants who had some knowledge on topics such as puberty, contraception, sexually transmitted infections (STIs), and the significance of informed decision-making within the realm of adolescent sexual health were purposively selected for the interviews in the schools and communities while policymakers and non-governmental agencies were selected based on their roles and relevance to adolescent sexual and reproductive health. They include State Ministry of Health, State Ministry of Education, State Ministry of Information, State Ministry of Youth and Sports Development, State Ministry of Women Affairs and Social Development, State House of Assembly, State Universal Basic Education Board, State primary health care development agency, civil society organizations, religious and traditional leaders (Table 1).

### Instrument and data collection methods

The study instruments included pre-tested topic guides for in-depth interviews, (IDI) and focus group discussions (FGD) which were developed by a team of qualitative research experts (Supplementary file 1). They were carried out to gain an in-depth understanding of the factors enabling and constraining the adoption of the intervention. The interviews were carried out by experienced researchers in qualitative study in English language. The data collection exercise was led by COM with support from AO and ICA. A total of 18 FGDs and 30 IDIs were conducted, based on the need to attain data saturation. The distribution of the interviews among different groups is shown in the table below.

**Table 1:** The distribution of the interviews among different groups

Policymakers and boundary partners – 16 IDIs	Health service providers and supervisors – IDIs (14)	Parents, community leaders and adolescents - FGDs (18)
State Ministry of Education (SmoE) (1) State Ministry of Health (SmoH) (3) State Ministry of Information (SMOI) (1) Legislator (1) State Primary Health Care Development Agency (1) Ebonyi State Ministry of Youth and Sport Development (ESMOYSD) (1) Media personnel (2) SDGs (1) Traditional rulers (2) Religious leader (1) Non-Governmental Organisations (NGOs) (1) Civil Society Organisations (CSO) (1)	LGA ASRH focal officers (6) – IDIs PHC workers (OICs) – 2 IDIs Patent Medicine Vendor (PMV)s – 2 IDIs Community Health Workers (CHWs) – 2 IDIs Local Government Area (LGA) admin secretaries (2) – IDIs	Teachers/Principals/GC (6) Male parents (1 FGD) Female parents (1 FGD) Male community leaders (1 FGD) Female community leaders (1 FGD) Male in-school adolescents (2 FGDs) Female in-school adolescents (2 FGDs) Male adolescents in-community (2 FGDs) Female adolescents in-community (2 FGDs)

*Boundary Partners refer to individuals or groups who actively contribute to, and with whom the project or program can engage to drive positive change such as the officers in charge of primary health centres, midwives, adolescent focal persons, and local government reproductive health officers

The objective of the interviews was well explained to the participants before the interviews commenced. All interviews were audio recorded with the permission of participants. Hand-written notes were also taken. At the end of each interview, audio files were well-labelled and saved on a secure computer.

### Data analysis

**Fig. 2:**
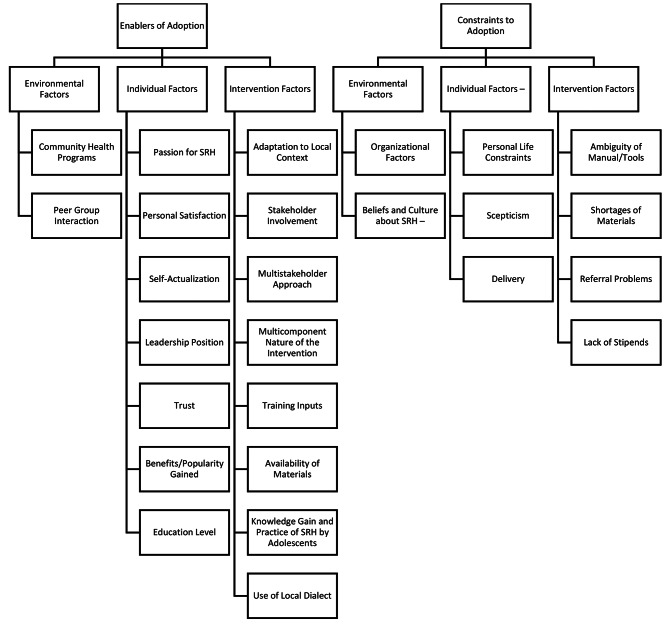
Code tree

All audio files were transcribed verbatim. Three (AO, ICA and COM) of the researchers were involved in data analysis. Under the guidance of COM, the lead researcher, the team utilized the coding reliability approach, a type of thematic data analysis. The following broad themes were defined as the coding categories: environmental factors, individual factors, and intervention factors. These themes were chosen based on their relevance to the research objectives and the theoretical framework guiding the study. The researchers read through six transcripts and developed a codebook, guided by the research objectives. They were merged and imported into Nvivo tool for qualitative data analysis. Nvivo facilitated the systematic organization, retrieval, and analysis of the coded data. During coding, coders searched for evidence or examples that fit each of the coding categories. The code tree is captured in Fig. 2.

### Findings (table 2)

**Table 2:** Summary of findings

Theme	Enablers	Constraints
Environmental	The intervention appeals and aligns with stakeholders’ goals and aspirations.	The increased workload of providers.
	Using adolescents as advocates influenced the adoption.	Transferring trained providers
		Belief about the timing of SRH communications with young people.
		Fear of delivering SRH in religious settings.
Individual	The intervention appeals and aligns with individual values and morals.	Interference with personal life.
	It brought direct and indirect benefits for community advocates	Scepticism of the importance and impact of SRH issues
		Poorly trained providers
Intervention related	Adapted to local contexts.	Technicalities and ambiguities in the manual
	Multi-stakeholder engagement.	Shortages of materials and tools
	Use of the multicomponent approach	Conflict of interest with project objectives.
	Active and recognized roles assigned to stakeholders	Poorly recruited advocates and providers
	Training of advocates.	Non-provision of stipends to providers and advocates.
	Supply of materials needed for awareness.	Profit orientation of PPMVs

### Enablers of adoption

#### Environmental factors

The environmental enablers to the adoption of the intervention include community and background or external factors like existing community health programmes and those spreading them. It also includes peer interaction in the community that improves knowledge and the adoption of the intervention.

#### Existing community health programmes

The intervention took place in communities where there are existing programmes addressing different health challenges. Stakeholders in health who have also been working in these communities adopted the project from inception because they think that it appeals to them and could help them actualise their objectives in these communities.

Some stakeholders, like the media that are outside the health sector, believe that the intervention aligns with existing media campaigns to promote the health and well-being of adolescents and young people. They remained open to the adoption of the intervention because they also saw the intervention as a novel way of addressing SRH issues. The supporting quote is shown below:

“Well of course as a media person, it has been part of our procedures and advocacy before the program came. That is why I said that immediately we saw you we did not hesitate to embrace you because it is already part of our programme. This is because we believe in the welfare and wellbeing of every segment of the society, especially the young ones” (IDI, Director Media Personnel).

#### Peer group interaction in the community

The intervention facilitated critical discussions and the exchange of ideas about SRH among peer groups which promoted positive attitudinal change. The positive response observed among the adolescents when their fellow adolescents told them about the benefits of SRH is evidence that peer influence had an impact on the adoption of intervention by adolescents.

Adolescents felt more comfortable when their peers gathered together in groups to talk about SRH matters. Below is a supporting quote:

“Me I will say that it is not difficult because when we are in a group like classmates and we are talking about sex, you know we teenagers, we like to hear such, they will bring their seat to listen to it. It is not difficult. Before you know it, everybody will be like they are talking about sex let’s go and listen to them, before you ‘know it they will form a group and you start telling them” (Female Adolescent in-school, Afikpo South).

### Individual factors

Individual factors identified across various stakeholders as enablers of adoption of the SRH interventions include passion, personal interest in SRH and satisfaction, priority setting/proper planning, teaching/leadership position, trust, benefits/popularity gained, and education.

#### Interests/ passion for SRH

Some of the stakeholders had personal interest in the SRH intervention and volunteered to participate in the intervention without looking forward to monetary gains. The satisfaction and the desire to see things work served as a motivation that enabled the adoption of the intervention. The following quote captures it this way:

“So the point is that I don’t have a single regret, if I ever have a regret it is something just like I said before, I volunteered here, it’s not like I wrote an application and am doing, within the work am doing gives me satisfaction and it’s not about money and let me just say this also, if you talk about money I cannot remember any of your events that you didn’t give money as a matter of fact but I don’t like to quantify my service and my personality cash wise but if you must mention that because the society we work in a lot of people will start with any more money for us but you can see that some of us like I said I am an employer in the NGO” (CSO representative).

“I don’t give them any money but I try to carry them along in whatever thing I am doing. In planning, we plan it together and you know this work is what we do with passion and the ones I am working with so far have the same passion” (NGO informant).

#### Self-actualisation

The intervention brought personal benefits to community advocates, both in skill development or self-actualisation. The training on SRH was noted to have improved the capacity and knowledge of individuals who were already teachers or leaders. The repeated engagements with members of the community increased advocates’ popularity and brought a sense of fulfilment to them. One of the health workers expressed how encouraged and elated she felt when recognised by community members because of the work she does:

“I benefited a lot, it encourages me and boost my moral in doing the work because this work has made many people to know me in our community, it has exposed me, making me to be popular in the community, many women and girls use to come to me asking for certain questions about their sexual lives, they even call me nurse now in my community” (Service provider, Izzi, FGD).

### Intervention factors

Factors related to the intervention that are enablers include adaptation of training manual to local context, easy readability and understanding of the manual, stakeholder involvement, multi-level and multidisciplinary nature of the intervention, multi-component nature of the intervention, supervision, availability of materials for field work, knowledge gained and practice of SRH by adolescents, and us of local dialect.

#### Adaptation of intervention to local context

One of the factors highlighted by the policymakers that made the intervention appealing is that it was adapted to the local context. The intervention and intervention materials were designed to accommodate community peculiarities and include local actors (i.e., religious persons, traditional leaders, parents, community teachers and young adolescents) who are able to drive positive change in young people. Their recognition and inclusion influenced the adoption of the intervention from inception and recorded interesting success.

In addition, training and awareness creation at the community level were carried out using the local dialect of the people. This improved comprehension and understanding of SRH. Using the local dialect also had a way of attracting the attention of the people and making them comfortable. They found it more interesting and felt at home knowing that the person passing the message across is one of their own and not a stranger. For example:

“It is easy because we were taught with our language and we understand it very well. Had it been they used English language to teach, you will understand some but will not understand some, using our dialect made it very easy for us to teach with” (Female Community Leader Agalegu, FGD, 03).

#### Multistakeholder approach

The intervention considered stakeholder involvement was also pointed out as an enabler to its adoption. Stakeholders at various levels (state, local government, facility, and community level) were involved in the intervention. In addition, stakeholders from different sectors and organisations such as education, media, health, information, women’s affairs, non-governmental organisations, civil society organisations, etc. were involved. One of the stakeholders reported that carrying the government agencies and other sectors along right from the planning phase played an important role in adoption of SRH.

“Ok thank you, let me re-emphasize again that the strategy you adopted because it’s one of your strategies because when you get the stakeholders involved in any activity or program you want to execute, it goes a long way because if the stakeholders are not aware that program is bound to fail” (Director, SMOH).

The multistakeholder approach also employed a multicomponent approach, which implies engaging stakeholders to contribute at different levels. Apart from training of the top stakeholders, individuals were trained at various levels including at the health facilities, schools, and community levels. This approach was noted to be instrumental in the adoption of SRH intervention because it included people working to achieve a common goal. Some supporting quotes include:

“.….I think the strategy is very fine because as I am the x in the state, I can’t be everywhere so if people are trained at each session or each level at the local Government, community level or facility level, they will also be of good use to talk about this adolescent reproductive health [….]so they will also help to train these people, expose them to things that are good. So, training the health workers or volunteers is also very relevant and important (Healthcare executive at SPHCDA).

Furthermore, these various stakeholders were involved in the development of the manual and this made it possible to capture diverse opinions that made adoption easy.

“The way and manner how these manuals were developed; FANTASTIC because I remember that all the major stakeholders were brought, you say your own, this other person say your own and we harmonize. It’s a very good strategy” (Director, at SMOH).

#### Training inputs

The training improved knowledge and skills to deliver SRH messages to adolescents. As for adolescents, it improved their confidence in discussing the topic with their peers and helped them recognise divergences on SRH issues. Adolescents reported adopting what they learnt and have equally made positive impact in the lives of their friends and siblings:

“What helped me to be able to teach others is that I have put the teachings into practice, that is why I decided to teach my friends and my siblings, I watch them as they put it into practice, and I am very happy that they are doing according to what I taught them” (Female adolescent in Community, Nwofe, FGD, 05).

In addition, materials and tools for fieldwork were given to some service providers, adolescents, teachers, and community leaders who participated in the training. The materials and tools included branded T-shirts, face caps, hand bands, posters, and handbills, designed to promote SRH. They enhanced the adoption of the intervention because it created more awareness and also provided avenues for the topic to be discussed. According to the respondents, the materials were instrumental in raising the topic of SRH for discussion. Some quotes are:

“One of the posters were given to me and I pasted at one chemist shop and I told the owner, if anybody has problem and wants to see the person that pasted this, she should call me and they use to call me after going through it. I posted it there because I know that people always go there to buy medicine. So it is also an avenue to tell people about the services”(Service oprovider, FGD, 06, Izzi).

“I used my own very well, I use to put on my own T-shirt even when I am going to the church and people will be asking me questions about it, I will teach the person there and then. Some people will learn it there on the spot. I also dress on my T-shirt whenever I am going to the office, people ask me questions and I teach them” (Female community leader FGD No 04, Agalegu).

### Constraints to the adoption of the intervention

#### Environmental factors

##### Organisational factors

Some organizational factors interfered with the decision of health workers to adopt the intervention by health workers. The OICs were expected to collect monthly data about referral cases but they would have to combine this task with other engagements. This affected the understanding of the project’s objectives and the delivery of quality SRH services. They also expressed concern with documenting the cases referred to them. Therefore, the willingness to try these new experiences or consider these new ways of meeting SRH needs of young people may increase workload or interfere with existing projects.

“I mean the health workers, they have a lot to do at this period, so, I cannot say the captured everything they expected them to do, because they are running helter-skelter, doing other partners work, they are too many projects that are going on now (IDI, FP, Female, 53 years-old)”.

“It is easier [SRH counselling] but in terms of documentation, there are a lot of tables and calculations in the summary part of it, at times you skip some months without written the summary because there are a lot of registers from other NGOs, kike, NHIS, HIV, GBV, TB is also there, there are a lot of registers to sum at the end of every month, so a times we skip because data collection tools are much with us” (FGD, OICs, Abakaliki).

Secondly, health workers who were part of this research programme and received training were transferred, making it difficult to ascertain their contributions to the project objectives. Some of them even left with the training manuals and other materials provided during the period. For example,

“Those people that were transferred, they are health workers, and another person was transferred and it’s like he left with the manual and another person that took over from her was asking for her won copy of the training manual.’ (IDI, Coordinator of reproductive health, female)”.

##### Believe and culture about SRH

The intervention took place in areas where culture influences communications about SRH. While the core objective of the project was to suggest and encourage positive behavioural changes among adolescents, some receivers of the intervention do not appeal to this due to deep-rooted beliefs about SRH. This new practice interfered with the belief about SRH issues. It was noted that some adolescents and parents, especially in rural areas, think that SRH communications should be limited to those who are married:

“Some of the challenges, all cultural barriers, that some of our parents and people there in the community, sometimes will stop these children from accessing the services, they will prevent or stop them from coming” (FGD, OIC PHC, Abakaliki).

Additionally, parents who are advocates reported encountering challenges while delivering SRH in religious settings. They reported that discussing SRH among adolescents in churches affected how they were perceived by others and in extreme cases, some of them reported that it affected their businesses. This is because the discussions about sex and sexuality were perceived to be secular and not religious inclined:

“It is difficult in some ways because I teach in the church, it is not easy for someone to come to the church and begin to talk about SRH, people will conclude that you do not have fear or shame to talk about such thing in the church, they will begin to see you as an irresponsible person” (FGD, Female parents, Nwofe).

“Yes, I think, like two of them, the one I told you about, the sons of pastor, may be because their religion did not accept what I preach to them, they stopped coming to buy something from me, so I have lost customer from them” (FGD, PMVs, Abakaliki).

#### Individual factors

##### Personal life

Adolescents who participated as mentors recounted how difficult it was to keep up with the project because it raises constant scrutiny about their personal lives. It was particularly difficult for those who have been seen in the past engaging in what they are currently teaching. Somehow, this demotivates them from adopting the initiative. For example:

“It is not easy to teach because teaching has affected most of my lifestyle. If someone sees me when I am following a girl, the person will not be willing to listen to me whenever I am talking, they will say “did I not see you with a girl the other day?” It is not easy to teach” (FGD, Adolescent boys, Agalegu).

##### Scepticism

Certain parents express scepticism regarding the program’s influence on their children. They hold the belief that regardless of the introduced programs, children will follow their own inclinations, deeming any efforts to positively impact their sexual and reproductive health (SRH) as futile and a waste of time.

“One man specifically said that the best thing to do “is to leave them” but I asked to leave your children like that and he said “will I tie them with ropes?” and that is it” (IDI, Vice principal, Ezza South).

##### Poor delivery of SRH

We gathered that some of the health workers do not adhere to the trainings about how to deliver quality SRH services. It was reported that:

“Health workers approach (in a bad tone and frowned face) it’s another thing all together but now we are dealing with that because it has been scaring, many people run away from going to health facility or even hospitals but I think as primary health care is concerned, we are seriously dealing against it” (IDI, EBSPHCDA, Female).

#### Intervention-related factors

##### The ambiguity of manual/tools

The implementation process made use of training the trainer’s approach, leading to the training of primary health workers, community health workers, and patent medicine vendors. However, some of the focal persons who participated in the training complained that there were technicalities in the delivery that made comprehension difficult. Specifically, the manuals/tools were said to be ambiguous and with small font sizes, making them difficult to understand and discouraging to read.

“That is where the problem lies, it is difficult for us to understand, because the grammar used was very high, some of us didn’t go to school like that, (she laughed) at times, we don’t know where to start, some of us don’t even read it, some of the health workers found it difficult to understand it, next time you make it simpler and more interactive” (IDI, FP, Female, 53 years-old).

“The manual was difficult to use especially for some of us that are old because I could not read the characters in the manual. They should endeavour to bolden the characters in the manual in case of next time” (FGD community leaders, male Akalagu).

##### Shortages of materials

There was quite a number of complains of the shortage of materials and tools. All the advocates reported that the flyers and posters were not enough for them to drop with community members who might be interested in reading more information. The shirts that were distributed at the beginning of the intervention was said to have promoted positive reaction but later, it worn off and was not replaced. This discouraged members from participating in outreach programmes at the later stage of the intervention. See illustrative quotes:

“No, because if there is an arrangement to extend the teaching we don’t have materials to do that, like going to the villages, market square, we don’t have materials for doing that and too amongst the students some of the have overused their T-shirts that write ups are no more showing on the shirts and if you tell to wear it that we are going for evangelism to preach “healthy life” they will their own is dirty and old and the only thing that can attracts audience is something like that” (IDI, Vice Principal, Male, Amuzu).

##### Referral problem

The implementation process also specified ways through which adolescent SRH needs would be met. Based on this, PMVs and schools were required to refer adolescents with SRH issues to OICs in their communities. The OICs, on their part, were trained to provide expert opinion that addresses adolescents’ SRH concerns. However, one OIC who also double as the focal person of her local government explained that this was not the case as many PMVs did not meet up with this demand. It was reported that the referral process is still problematic.

“The only place that needs to be stressed is the referral process, the PMV dealers, doesn’t refer adolescents with SRH issues to us. Also, the schools that were trained have not been referring students to us. I have never received any referral from PMV” (IDI, FP, Female, ND).

The challenge with the referral process could have been induced by the method used to identify and include PMVs in the trainings. It was gathered that some PMVs who participated in the project do not practice at the intervention sites and as such could not provide the needed services or act within expectations of the project objectives. For illustration:

“Most of these people selected were not in that locality, the place of intervention, do you understand, so, yes, they are working in various places but on the situation whereby no PMVs were selected on the intervention community, it has a big question mark, so, because in Agbaja as a whole, no PMV was selected, even though, Izzi Unuhu, the larger Community, they picked from it but the core intervention area, no PMV was picked, they have 3 CHWs but no PMV’s, so that was an implementation gap” (IDI, FP, Female, 53 years-old).

##### Lack of stipends

Some health workers, PMVs, parents and teachers were trained to support adolescents who have ASRH issues. These advocates were supported by supervisors who would help them meet the projects objectives and proffer solutions to any challenges. To achieve this, the supervisors may need to frequently visit the communities and the facilities, as the case may be. One problem that was consistent in the data from supervisors is that advocates of ASRH are demotivated due to lack of stipends. This was said to have interfered with their openness to the interventions because they expected to be compensated for their work. For illustration:

“They are complaining of how they work from month to month and they are not being paid, no stipends are giving to them. One of the patent medicine officers is also complaining on the same thing. It’s really difficult for them and this is a program they have gone for and yet nothing has been done” (IDI, FP, Female, 41 years-old).

The requirements for PMVs to provide SRH services within the project’s objectives interfere with their profit orientation. During the training periods, they were constantly drawn to their business that they could hardly immerse themselves in the project. Additionally, they rarely avail themselves to addressing adolescents SRH needs due to the desire to increase sale. For example:

“That is where am worried, it is not enough, PMV’s is business-oriented, they wouldn’t have patient enough to teach those people, because of their time. I think it is appropriate, just that it could not reach all of them, because of time constraints” (IDI, FP, Female, 53 years-old).

“Sometimes, especially in the evening, if you have customers that came to buy medicine and there are group of them you are already counselling, there will be saying that am busy discussing with others and I don’t want to attend to them, so, is one of the challenges, and you know I cannot leave my customer and you cannot also allow them to go, because if they go, they may not come back again” (FGD, PMVs, Abakaliki).

## Discussions

The study found that stakeholders responded positively to the implementation of community-embedded adolescent health intervention and demonstrated their readiness to adopt the practice. During the project design phase, the researchers held several consultative meetings and co-production workshops to identify and include all stakeholders and get their buy-in and contributions to the project design. The stakeholders’ input led to changes in the original intervention design in ways that met most of their needs. This approach ensures that the inputs of stakeholders reflect the local contexts [27]. As the findings show, meeting the needs of stakeholders speedily enabled the adoption of the intervention at the earliest phase. This is probably because health policymakers and the media saw it as an avenue to accelerate the state’s agenda on the health and well-being of young people. In a study evaluating the use of result-based financing to improve maternal and neonatal health, it was reported that involving key stakeholders at the earliest phase of intervention promoted adoption and helped in overcoming resistance [15].

The implementation design also enabled the adoption of the intervention by accommodating community peculiarities. In a study in Zambia, it was found that the success of SRHR interventions rests on their ability to consider community context [28]. In this study, the implementation design had manuals and tools adapted to local contexts while training and awareness at the community level were carried out in local dialects. Also important at the design stage is the supply of branded t-shirts, face caps, and other materials to community advocates. This raised their commitment even as they utilised the materials as proof of their involvement in larger community development initiatives.

The project was also adopted because it aligned with the stakeholders’ interest in SRH. Those passionate about an improved SRH of young people volunteered and demonstrated ownership at the beginning of the project. This also brought a sense of fulfilment to those already at the forefront of making critical contributions to adolescents’ SRH. Indirectly, it serves as a means of self-actualisation even as stakeholders pursue their interests which also align with the project objectives. As shown in a similar study, stakeholders’ interests may be influenced by the level of power they wield to make changes [17]. Thus, implementers of ASRH projects may witness early adoption if their project objectives align with powerful stakeholders (these are key players who have substantial impact on decision-making processes as regards SRH at various levels).

On the other hand, the adoption of the intervention was constrained by the demands placed on providers. They experienced heavy workload while combining their tasks with their primary assignments. As a worker in an organisation, it was expected for them to respond to the organisation’s need before meeting the requests of the project. In this study, the supervisors complained that they could not meet their demands from the early stage of the intervention due to primary assignments at the facility level. One study has shown that in regions experiencing human resources crises, combining the project’s task and primary responsibilities may result in an increased workload [29].

Similarly, adolescents who participated as mentors recounted how difficult it was to keep up with the project because it raises constant scrutiny about their personal lives. This served as a constraint because they were constantly judged in ways that demotivated them. Hence the drop in zeal and motivation to continue to try the initiative.

The interplay among culture, religion and societal norms also contributed as a constraining factor to the adoption of the intervention. The beliefs about SRH created resistance to the positive behavioural changes expected of those who received the intervention. Discussing SRH topics in churches is viewed as a secular activity, rather than religiously inclined. Likewise, parental restrictions and community stigma reinforced the perception that SRH discussions should be limited to only married individuals. In another study, parents only communicate health issues with their children if they perceive that their lives are under immediate threat [30]. This emphasises the importance of culture and context in the adoption of SRH intervention.

The manuals, tools and materials were not sufficient for use and distribution throughout the implementation. Our findings show that there was low motivation to be part of the outreach because of the lack of materials. These materials are important because they announce the presence of these stakeholders and give them a sense of togetherness. However, when it is not available, stakeholders become demotivated and refuse to participate in awareness campaigns.

### Recommendations for future interventions

The prevailing social attitudes and practices at the individual, community, school, and health facility levels often present major barriers to adolescents seeking SRH services. In this study, we have shown that stakeholders can adopt a complex intervention that supports adolescents in ways that enhances communication and strengthen bonds with teachers, healthcare providers, family members and the community. Much like the CERCA project which utilised a community-embedded intervention for reproductive healthcare for adolescent girls, this intervention encouraged and enabled discussions and reflections about SRH services at the community level [3]. However, implementers may need to up their engagement process to reduce the effect that is due to norms and religious beliefs. Also, interventions may be difficult to adopt when receivers have vested interests that may interfere with the project objectives. Hence the need to design projects with the consideration of stakeholders’ interests. Finally, future studies may want to consider the role of incentives, including monetary, in enabling or constraining the adoption of community-embedded interventions. This is considering the argument that incentives alone cannot guarantee systems adoption [31, 32].

### Limitation

Despite these findings, the study has several limitations. First, many types of evaluation outcomes exist but this study narrowed to adoption. While this approach provides valuable insights into the uptake of the intervention, it does not capture the full picture of the intervention’s impact or effectiveness. Future studies may need to explore these, including acceptability and appropriateness from the consumers’ or receivers’ point of view. The study may also benefit from a quantitative assessment of the issue.

## Conclusion

There was widespread adoption of the community-embedded reproductive health intervention from early phase of the implementation. At the later phase, however, hesitancy was noted mainly due to the non-incentivisation of community advocates. Getting the right stakeholders to work for the progress of intervention yields many good results. It enables the adoption of the approach especially when stakeholders are engaged appropriately and at the right time.

### Electronic supplementary material

Below is the link to the electronic supplementary material.

Supplementary Material 1

## Data Availability

The dataset for the study can be obtained from the corresponding author upon reasonable request.

## Abbreviations

ASRH: Adolescent Sexual and Reproductive Health
LGA: Local Government Area
CTR: Council of Traditional Rulers
IDI: In-depth Interview
FGD: Focus Group Discussions
PMV: Patent Medicine Vendors
OICs: Officer-in-Charge
EBSPHCDA: Ebonyi State Primary Healthcare Development Agency
CHW: Community Health Workers
