# Supplementary material for: Factors influencing adoption of sexual and reproductive health intervention for adolescents in Ebonyi, Nigeria
Source: BMC Health Serv Res. 2024 May 19;24:643. doi: 10.1186/s12913-024-11103-y (PMC11102607; doi:10.1186/s12913-024-11103-y)
Supplement: Supplementary file 1 — Supplementary Material 1 [file 12913_2024_11103_MOESM1_ESM.docx]

**Addressing unmet SRH needs of adolescents using community-embedded interventions in Ebonyi State**

**Evaluation of Advocacy Interventions**

**In-depth interview guide for policymakers and boundary partners**

Introduction, purpose and procedure

I am a trained data collector from Health Policy Research Group University of Nigeria Enugu Campus and we are working with Ebonyi State government to evaluate some interventions/programs that were implemented to address the Sexual and Reproductive Health needs of adolescents in the State. .

The aim of this interview is to get your views on whether and how these interventions/programs made an impact of adolescents in Ebonyi state Nigeria.

I am particularly interested in your experiences during the implementation of the interventions in your community/LGA/State, as well as your recommendations on how to improve and sustain the quality of SRH services and information for adolescents. The information you provide in this interview will be treated as confidential and your participation will be anonymous.

With your permission, I would like to record this interview to make sure I accurately capture our discussion. This interview will last about 45 minutes.

***Interviewer turn on the audio recorder and say,***

*“This is an in-depth interview with* [position/office or respondent] *in* [name of agency/ministry/organization]. *The interview is taking place at* [venue] *on* [day, dd/mm/yyyy]. *The start time is* [hh:mm am/pm] *and the interviewers are* [names of interviewer and note-taker]*”*

***Before you start the interview,***

- ***Ask the participant to introduce himself/herself including their age, office/position, and their primary roles in the office. (****While this is going on, the note-taker should complete the participants’ information form****)***

***At the end of the interview,***

**Thank the participant for his/her time.**

**Then turn off the audio recorder after stating the time the interview ended,** *“The discussion ended at [hh:mm am/pm]”.*

## **Discussion**

1. What are your views about the advocacy intervention that was implemented by Health Policy Research Group and government agencies in Ebonyi State to address the SRH rights and needs of adolescents?

**Prompt respondent to reflect on the advocacy strategies they participated in with respect to (i)** strengthening inter-sectoral collaboration in the provision of quality and comprehensive SRH information and services to adolescents; and **(ii)** institutionalization of comprehensive sexuality education (CSE) in secondary schools and non-formal education settings

**Probe for –**

- Relevance and appropriateness of strategy - *Was the strategy relevant? Was it appropriate? What makes you think so?*
- Fit of strategy/Burden – *How well did the implementation fit the existing programs/plans for ASRH? How well did it fit your own roles and responsibilities? How well did it fit your own schedule and plans? Why makes you say so?*
- Opportunity cost – *What things, opportunities or values (such as office/community/family or personal engagements) did you have to give up in order to organize and/or deliver the interventions* (specify)*?*
- Ethicality - *How has participating in these interventions* (please specify) *impacted on your personal beliefs and values? How suitable are these interventions to the beliefs and norms of your community?*
- Effectiveness of the strategy – *To what extent has advocating to policymakers, traditional rulers, community leaders and gatekeepers,*
  - *Improved inter-sectoral collaboration in the provision of quality and comprehensive SRH information and services to adolescents? How is this so?*
  - *Influenced (facilitated) the institutionalization/entrenchment of CSE in secondary school curriculum? How is this so?*
  - *Facilitated community support in reviewing/revising the socio-cultural barriers mitigating adolescents’ SRH rights and needs?*

1. Since after the advocacy interventions for strengthening inter-sectoral collaboration in the provision of quality and comprehensive SRH information and services to adolescents, what has been your experience with partnering with other sectors or agencies (including individuals and groups) to promote the SRH rights and needs of adolescents? **Probes:**

- Who are those that you have partnered with? *(****Prompts:*** *other government agencies; NGOs; CSOs; donor agencies; implementing partners; etc).* Why did you decide to partner with them?
- What were the roles of the different actors in the partnership?
- What kind(s) of activities did you provide through/in these partnerships? *(****Prompts:*** *health services – counselling, referral, products etc; health information – awareness creation; etc)*
- Did you experience any difficulty while partnering with other agencies, or was it a simple task? (**Probe**: Why do you say it is difficult or simple task?)

**Interviewer please repeat question 2 (including probes) this time focusing on institutionalization of comprehensive sexuality education (CSE) in secondary school curriculum**

1. How do you intend to carry on with the advocacy interventions?
   - Promoting (or sustained advocacy for) the entrenchment of CSE in secondary school curriculum to ensure sustained delivery of quality SRH information to adolescents?
   - Promoting (or sustained advocacy for) inter-sectoral collaboration in the provision of quality and comprehensive SRH information and services to adolescents?

***Probes:*** *Are you willing and open to carry on? Do you think the tools you have been given are sufficient? What makes you say so?*

1. What are your suggestions for how to sustain advocacy for adolescents’ SRH rights and needs in Ebonyi State? (What should be done and how can it be achieved?)

## **Addressing unmet SRH needs of adolescents using community-embedded interventions in Ebonyi State**

## **Evaluation of Health Information Interventions**

## **FGD Guide for Adolescents that participated in the community campaign sessions**

### **Introduction, purpose and procedure**

Hello. My name is ___________________ and I am a trained data collector from Health Policy Research Group University of Nigeria Enugu Campus. We are working with Ebonyi State government to evaluate some interventions/programs that were implemented to address the Sexual and Reproductive Health needs of adolescents in the State.

The aim of this interview is to get your views on whether and how these interventions/programs made an impact of adolescents in your community and school.

I am particularly interested in your experiences during the implementation of these programs in your community, as well as your recommendations on how to improve and sustain the quality of SRH services and information for adolescents.

The information you provide in this interview will be treated as confidential and your participation will be anonymous.

With your permission, I would like to record this interview to make sure I accurately capture our discussion. This interview will last about 60 Minutes.

***Interviewer turn on the audio recorder and say,***

*“This is a focus group discussion with* [in-school or out-of-school] *adolescent* [boys or girls] *in* [name of community]. *The FGD is taking place at* [venue] *on* [day, dd/mm/yyyy]. *The start time is* [hh:mm am/pm] *and the interviewers are* [names of moderator, note-taker, observer etc]*”*

***Before you start the discussion,***

- ***Ask each participant to introduce himself/herself including their age, grade in school or trade/skill being acquired, employment status and type of work done. (****While this is going on, the note-taker should complete the participants’ information form****)***
- ***Set the ground rules for the discussion with participants***
- ***Assign codes to each participant and ask them to mention their code when they want to speak***

***At the end of the discussion,***

**Thank the participants for their time.**

**Then turn off the audio recorder after stating that, *“The discussion ended at* [hh:mm am/pm]*”.***

## **Discussion**

1. What is your view about the adolescent SRH awareness campaign/sessions that was implemented in your community? (***Probe:*** *Why do you think so? What is the reason for that?*)

- In your opinion, how important was it to organize this awareness campaign in your community? How was it relevant? How helpful has it been in providing appropriate SRH information to adolescents?
- How did participating in the sessions make you feel? **Prompts:** emotions, relevance, value, etc

*(****Probes:*** *Why do you feel this way? What makes you feel this way?)*

- How did the program make an impact in your community?

*(****Probes:*** *Why do you say so? Which groups were particularly impacted? In what ways were they impacted? Could you share some examples?)*

- How do you feel about knowing the trained health workers, school teachers and peer educators in your community? **Prompts:** emotions, relevance, value, sense of community/support etc.

(***Probes*:** *Why do you feel this way? What makes you feel this way?*)

- - What is your relationship with them? *(****Probes****: Why do you think this is so? What has hindered or sustained this relationship? How has this relationship helped you in championing adolescents’ SRH in your community?)*

1. Since after participating in the community campaign on adolescent sexual and reproductive health, what is your outlook/view/opinion on the sexual and reproductive health rights (SRHR) and needs of adolescents? ***(Probes:*** *Why is this so? Could you explain more?)*

- How did participating in that campaign influence the way you think about the SRHR of adolescents?
- What are you doing differently? And how? (Prompts – in terms of SRH behaviour, communication with other adolescents, awareness creation, etc.)
- What kinds of changes are you making or have you made? And how? (Prompts – at home, in the community, in school, in work place, in social groups, etc)

1. What has been your experience with sharing the information you received during the campaign with other people in your community? **Probes:** *With whom have you shared the information?*

- *What prompted/made you to share the information with them? What did you tell them?*
- *How did they receive the information? Were they interested in the discussion or dismissive or argumentative? How did you keep them interested/engaged in the discussion?*
- *Did they have any concerns/questions that you were unable to answer? How did you handle those?*
- *Do you find it difficult engaging your peers on SRH discussions or is it a simple task? Why do you say it is difficult or simple task?*
- *How suitable are these SRH discussions to the beliefs and norms of the people in your community? Why do you think this is so?*
- *How comfortable are you to have these SRH discussions with other adolescents even if it is very different from your beliefs or theirs?*
- *What has enabled or made it easier for you to share SRH information with people in your community?*

1. What are your suggestions for sustaining SRH information to adolescents and parents in your community?

***Probes:*** *What should be done? How can it be achieved?*

## **Addressing unmet SRH needs of adolescents using community-embedded interventions in Ebonyi State**

## **Evaluation of Health Information Interventions**

## **FGD Guide for Adolescents that participated in the school-based interventions**

### **Introduction, purpose and procedure**

I am a trained data collector from Health Policy Research Group University of Nigeria Enugu Campus and we are working with Ebonyi State government to evaluate some interventions/programs that were implemented to address the Sexual and Reproductive Health needs of adolescents in the State. The aim of this interview is to get your views on whether and how these interventions/programs made an impact of adolescents in your school and community. I am particularly interested in your experiences during the implementation of these programs in your school, as well as your recommendations on how to improve and sustain the quality of SRH services and information for adolescents. The information you provide in this interview will be treated as confidential and your participation will be anonymous.

With your permission, I would like to record this interview to make sure I accurately capture our discussion. This interview will last about 60 Minutes.

***Interviewer turn on the audio recorder and say,***

*“This is a focus group discussion with* [in-school or out-of-school] *adolescent* [boys or girls] *in* [name of community]. *The FGD is taking place at* [venue] *on* [day, dd/mm/yyyy]. *The start time is* [hh:mm am/pm] *and the interviewers are* [names of moderator, note-taker, observer etc]*”*

***Before you start the discussion,***

- ***Ask each participant to introduce himself/herself including their age, grade in school or trade/skill being acquired, employment status and type of work done. (****While this is going on, the note-taker should complete the participants’ information form****)***
- ***Set the ground rules for the discussion with participants***
- ***Assign codes to each participant and ask them to mention their code when they want to speak***

## **Discussion**

1. What is your view about the school health club that was established in your school? (***Probe:*** *Why do you think so? What is the reason for that?*)

- In your opinion, how important was it to have a school health club? How was it relevant? How helpful has it been in providing appropriate SRH information to adolescents/students?
- How does being a member of the school health club make you feel? **Prompts:** emotions, relevance, value, etc. *(****Probes:*** *Why do you feel this way? What makes you feel this way?)*
- To what extent has the school health club contributed to dispelling myths & misconceptions about adolescents and SRH? How is this so?
- How has the school health club made an impact on the SRH of students in your school?

*(****Probes:*** *Why do you say so? Which groups were particularly impacted? In what ways were they impacted? Could you share some examples?)*

- How do you feel about knowing the trained health workers, school teachers and peer educators in your school/community? **Prompts:** emotions, relevance, value, sense of community/support etc.

(***Probes*:** *Why do you feel this way? What makes you feel this way?*)

- What is your relationship with them? *(****Probes****: Why do you think this is so? What has hindered or sustained this relationship? How has this relationship helped you in championing adolescents’ SRH in your community?)*

1. Since you joined the school health club, what is your outlook/view/opinion about the sexual and reproductive health rights (SRHR) and needs of adolescents? ***(Probes:*** *Why is this so? Could you explain more?)*

- How does being a member of the club influence the way you think about the SRHR of adolescents?
- What are you doing differently? And how? (Prompts – in terms of SRH behaviour, choices, communication with other adolescents, awareness creation, etc.)
- What kinds of changes are you making or have you made? And how? (**Prompts** – at home, in the community, in school, in social groups, etc)

1. How confident are you to provide SRH information to your peers and adults? (**Probes:** Can you perfectly engage your peers in a discussion on SRH matters? What is this so?

- Can you freely communicate with your parents or other adults on SRH matters?

1. What has been your experience with sharing the SRH information you receive from the school health club with your peers? **Probes:** *With whom have you shared the information?*

- *What prompted/made you to share the information with them? What did you tell them?*
- *How did they receive the information? Were they interested in the discussion or dismissive or argumentative? How did you keep them interested/engaged in the discussion?*
- *Did they have any concerns/questions that you were unable to answer? How did you handle those?*
- *Do you find it difficult engaging your peers on SRH discussions or is it a simple task? Why do you say it is difficult or simple task?*
- *How suitable are these SRH discussions to the beliefs and norms of your peers and community? Why do you think this is so?*
- *How comfortable are you to have these SRH discussions with other adolescents even if it is very different from your beliefs or theirs?*
- *What has enabled or made it easier for you to share SRH information with your peers in school or at home?*

1. Do you think that after providing SRH information to your peers in the community, they will be confident and comfortable to share with other people, given the sensitive nature of SRH?
2. What are your suggestions for sustaining SRH information to adolescents and parents in your community?

***Probes:*** *What should be done? How can it be achieved?*

**Thank the participants for their time. End the interview with, *“The discussion ended at* [hh:mm am/pm]*”.***

**Addressing unmet SRH needs of adolescents using community-embedded interventions in Ebonyi State**

**Evaluation of Health Information Interventions**

**In-depth interview guide for Parents and Community gatekeepers**

Introduction, purpose and procedure

I am a trained data collector from Health Policy Research Group University of Nigeria Enugu Campus and we are working with Ebonyi State government to evaluate some interventions/programs that were implemented to address the Sexual and Reproductive Health needs of adolescents in the State. .

The aim of this interview is to get your views on whether and how these interventions/programs made an impact of adolescents in Ebonyi state Nigeria.

I am particularly interested in your experiences during the implementation of the interventions in your community/LGA/State, as well as your recommendations on how to improve and sustain the quality of SRH services and information for adolescents. The information you provide in this interview will be treated as confidential and your participation will be anonymous.

With your permission, I would like to record this interview to make sure I accurately capture our discussion. This interview will last about 45 minutes.

***Interviewer turn on the audio recorder and say,***

*“This is an in-depth interview with* [position/office or respondent] *in* [name of agency/ministry/organization]. *The interview is taking place at* [venue] *on* [day, dd/mm/yyyy]. *The start time is* [hh:mm am/pm] *and the interviewers are* [names of interviewer and note-taker]*”*

***Before you start the interview,***

- ***Ask the participant to introduce himself/herself including their age, office/position, and their primary roles in the office. (****While this is going on, the note-taker should complete the participants’ information form****)***

***At the end of the interview,***

**Thank the participant for his/her time.**

**Then turn off the audio recorder after stating the time the interview ended,** *“The discussion ended at [hh:mm am/pm]”.*

## **Discussion**

1. What are your views about the small group awareness campaigns that were implemented in your community to address the SRH rights and information needs of adolescents?

**Prompt respondent to reflect on the elements of the awareness campaign –** training of parents, community leaders, and adolescents; and the distribution of handbills, fliers and posters.

**Probe for –**

- Relevance and appropriateness - *Was it relevant? Was it appropriate? What makes you think so?*
- Fit of strategy – *How well does it fit your own roles and responsibilities? How well does it fit your own schedule and plans? What makes you say so?*
- Opportunity cost – *What things, opportunities or values (such as office/ community/family or personal engagements) did you have to give up in order to participate in the awareness campaigns* (specify)*?*
- Ethicality - *How has participating in the awareness campaign impacted on your personal beliefs and values? How suitable is providing SRH information to adolescents to the beliefs and norms of your community?*
- Effectiveness of the strategy – *How has the campaign* (interviewer please specify the element) *affected the quality of SRH information that is provided to adolescents – including dispelling myths and misconceptions about SRH? How has it affected access to SRH information for adolescents?* *How has it affected communication between parents and adolescents?*

1. What are your thoughts on how the awareness campaigns were implemented?

**Prompt respondent to reflect on the elements of the awareness campaign –** training of parents, community leaders, and adolescents; and the distribution of handbills, fliers and posters.

**Probe for –**

- Availability of implementation manuals/protocols – *Was there an implementation manual/protocol for the intervention? Was this given to you and the other people that participated in the campaigns? Was the manual/protocol detailed and easy to understand?*
- Availability of other implementation tools (e.g. PowerPoint slides, group work guides, IEC materials, etc.) – *What other tools were used in implementing the campaigns? Were these tools given to you and other participants? Were they detailed and easy to understand?*
- Adherence to implementation agenda, manuals/protocols and tools – *To what extent have you used the tools you have given to provide SRH information to adolescents in your household and/or community? Which tools are being used and how are they being used? Which tools are not being used and why not?*
- How committed have you been to using the tools (specify) you were given provide SRH information to adolescents in your household and/or community? *Could you explain further? Please rate your commitment on a scale of 1-10 (1 being the least committed and 10 the most committed)?*

1. Since after participating in small group awareness campaign what has been your experience providing SRH information to adolescents and promoting the SRH rights of adolescents?

**Probes:**

- Who are those you provided SRH information to? *(****Prompts:*** *children, Spouse, other adolescents in the community, friends, neighbors, church members etc.).*
- Why did you decide to speak to them?
- What kind(s) of information did you provide? *(****Prompts:*** *prevention of unwanted pregnancy, counselling on sex-related matters, pubertal changes, contraceptives, risky sexual behaviors, forming relationships, parent-child communication, etc)*
- Did you experience any difficulty in providing the SRH information or was it a simple task? (**Probe**: Why do you say it is difficult or simple task?)

1. How do you intend to carry on with providing information about adolescents’ SRH rights and needs to ensure sustained delivery of quality SRH information to adolescents? ***Probes:*** *Are you willing and open to carry on? Do you think the tools you have been given are sufficient? What makes you say so?*
2. What are your suggestions for how to sustain provision of sexual and reproductive health information to adolescents and parents in your community? (What should be done and how can it be achieved?)

**Addressing unmet SRH needs of adolescents using community-embedded interventions in Ebonyi State**

**Evaluation of Health Services Interventions**

## **In-depth interview guide for health service providers**

### Introduction, purpose and procedure

I am a trained data collector from Health Policy Research Group University of Nigeria Enugu Campus and we are working with Ebonyi State government to evaluate some interventions/programs that were implemented to address the Sexual and Reproductive Health needs of adolescents in the State. .

The aim of this interview is to get your views on whether and how these interventions/programs made an impact of adolescents in Ebonyi state Nigeria.

I am particularly interested in your experiences during the implementation of the interventions in your community/LGA/State, as well as your recommendations on how to improve and sustain the quality of SRH services and information for adolescents. The information you provide in this interview will be treated as confidential and your participation will be anonymous.

With your permission, I would like to record this interview to make sure I accurately capture our discussion. This interview will last about 45 minutes.

***Interviewer turn on the audio recorder and say,***

*“This is an in-depth interview with* [position/office or respondent] *in* [name of agency/ministry/organization]. *The interview is taking place at* [venue] *on* [day, dd/mm/yyyy]. *The start time is* [hh:mm am/pm] *and the interviewers are* [names of interviewer and note-taker]*”*

***Before you start the interview,***

- ***Ask the participant to introduce himself/herself including their age, office/position, and their primary roles in the office. (****While this is going on, the note-taker should complete the participants’ information form****)***

***At the end of the interview,***

**Thank the participant for his/her time.**

**Then turn off the audio recorder after stating the time the interview ended,** *“The discussion ended at [hh:mm am/pm]”.*

## **Discussion**

1. What are your views about the SRH services that are being implemented in your facility to address the SRH rights and needs of adolescents?

**Prompt respondent to reflect on –** youth-friendly SRH service delivery (YFS); and supportive supervision of health workers

**Probe for –**

- Relevance and appropriateness of strategy - *Is the strategy relevant or valuable? Is it appropriate? What makes you think so?*
- Fit of strategy/Burden – *How well does the strategy fit the existing programs/plans for adolescent health in your facility? How well does it fit your own roles and responsibilities? How well does it fit your own schedule and plans? Why do you say so?*
- Effectiveness of the strategy – *How has this strategy* (interviewer please specify the strategy) *affected the quality of SRH services that are provided to adolescents? How has it affected the utilization of SRH services by adolescents?*
- Ethicality - *How has providing youth-friendly SRH services to adolescents impacted on your personal beliefs and values? How suitable is providing SRH services to adolescents to the beliefs and norms of your community? How do you view adolescents who come to access SRH services?*

1. What are your thoughts on how provision of SRH services for adolescents is being implemented in your health facility?

**Prompt respondent to reflect on –** youth-friendly SRH service delivery (YFS); and supportive supervision of health workers

**Probe for –**

- Capacity of health workers – *Who provides YFS in this facility? Why is this so? Have all health workers been trained to provide YFS? How was this done? What was used for the training? If No, why is this so?*
- Availability of guidelines/protocols – *Is there an implementation manual/protocol for YFS? Is this accessible to you and the other health workers in the facility? Is it detailed and easy to understand?*
- Availability of other implementation tools (e.g. job aides, posters, facility registers, etc.) – *What other tools are used in providing YFS? Are these tools available to you and other health workers? Are they detailed and easy to understand?*
- Adherence to guideline/protocols and other tools – *Are the guidelines/protocols and other tools adhered to and/or used during the provision of YFS? Please explain how this is done? Are the contents of the manual or tools* (specify tool) *fully covered during service delivery? Which tools are not used, and why?*
- How committed have you been in using the guideline/protocol and other tools (specify) in the course of providing YFS? *Could you please explain further? In what ways are you committed? Please rate your commitment on a scale of 1-10 (1 being the least committed and 10 the most committed)?*
- Supervision of health workers – *How have you benefitted from the supportive supervision that you have received in the provision of YFS? Please tell me more? How did it benefit you or not benefit you? What did you expect to receive/learn? In what areas were you impressed? What were your disappointments?*

1. In the past one year, what has been your experience providing SRH services to adolescents in your community?

**Probes:** *Has this been a difficult task or an easy one? What makes you say so? Could you please give some examples? How did you react when…? Did anyone else you know have a similar or different experience? Could you please tell me more about this?*

1. What changes have been made in the way that SRH services are provided to adolescents and young people in the primary health centre?

**Prompts:** What positive changes have been made? What negative changes have been made?

**Probes:** *Why do you think this is so? Could you please tell me more about these changes? How have they affected the care-seeking behaviour of adolescents? How have they affected the care-seeking behaviour of other young people in your community?*

1. In the past, did you feel comfortable providing SRH services to adolescents? Why is this so?

**Prompts:** *skills, confidence, tools, values/norms, beliefs, etc?*

**Probes:** *Why did it make you feel that way? How did it make you treat your adolescent clients?*

1. Do you now feel comfortable providing SRH services to adolescents and young people? **Probes:** *Why is this so? What/who makes you feel comfortable/uncomfortable? How does this happen?* *Could you share some examples?*
2. What challenges have you experienced in providing YFS to adolescent?

**Prompts –** *from adolescents, parents, community; availability of commodities; time and effort; workload*

**Probes –** *In what ways has this been a challenge? How has this challenge affected you and the services you provide? Could you tell me more? Is this also s challenge to your colleagues in this facility? Is this also a challenge to your colleagues in other facilities? How have you coped with or managed this challenge?*

1. How do you intend to carry on with routinely providing YFS to adolescents? ***Probes:*** *Are you willing and open to carry on? Do you think the training and tools you have been given are useful? What makes you say so?*
